# CT-guided percutaneous lung biopsy under calm breathing

**DOI:** 10.1515/med-2026-1533

**Published:** 2026-11-02

**Authors:** Meiru Wen, Ailin Chen, Zhiqiang Peng, Qimin Li, Songling Zhang, Shiliang Huang, Xiaofei Tang, Jing Liu, Meili Li

**Affiliations:** Department of Radiology, Ganzhou Cancer Hospital, Ganzhou, Jiangxi, People’s Republic of China; Department of Radiation Oncology, Ganzhou Cancer Hospital, No.19 Huayuanqian Road, Shuidong Town, Zhanggong District, Ganzhou 341000, Jiangxi, People’s Republic of China; Gannan Health Vocational College, Ganzhou, Jiangxi, People’s Republic of China

**Keywords:** syringe needle-assisted localization, respiratory motion, procedural feasibility, needle adjustment, pulmonary complications

## Abstract

**Objectives:**

To investigate the use of syringe needle-assisted localization to enable patients to undergo CT-guided percutaneous lung biopsy performed under calm breathing. This approach was developed to facilitate CT-guided lung biopsy in patients who have difficulty performing breath-holding maneuvers and to improve procedural feasibility under calm breathing conditions.

**Methods:**

A total of 200 patients who underwent CT-guided percutaneous lung biopsy were included and randomly allocated to either a syringe needle-assisted calm breathing group (n=100) or a conventional breath-holding group (n=100). Procedural outcomes, including puncture success rate, procedure-related complications (pneumothorax, pulmonary hemorrhage, hemothorax, chest wall hematoma, and air embolism), and the number of needle adjustments, were collected and compared between the two groups.

**Results:**

No statistically significant differences were detected in puncture success rate or complication rates. The calm breathing group showed a tendency toward fewer needle adjustments, although the statistical interpretation differed between the primary mean-based analysis and the non-parametric sensitivity analysis.

**Conclusions:**

CT-guided percutaneous lung biopsy under calm breathing with syringe needle-assisted localization was feasible in the present study and may be useful for patients who have difficulty cooperating with breath-holding instructions.

## Introduction

CT-guided percutaneous lung biopsy is an important minimally invasive method for obtaining pathological tissue from pulmonary lesions [1]. Accurate needle placement depends on stable lesion localization during the procedure. Because lung position changes with respiration, patients are usually instructed to hold their breath during needle insertion to reduce lesion displacement. Currently, common breath-holding methods include end-inspiratory breath-holding, end-expiratory breath-holding, and brief breath-holding during calm breathing; however, none of these methods can completely eliminate localization errors caused by respiratory fluctuations.

Fluoroscopy-guided biopsy achieves two-dimensional real-time monitoring but suffers from lower accuracy and still requires patients to hold their breath during needle insertion and biopsy [2]. CT fluoroscopy guidance allows for dynamic observation of the needle insertion direction, but it imposes high equipment requirements and exposes medical staff to radiation injury [3]. Studies involving laser-assisted CT-guided lung biopsy have suggested a reduction in needle adjustment frequency, while the influence of respiration on needle positioning remains unaddressed [4]. Although robot-assisted positioning technology can improve localization accuracy, unlike in static tissue models, robot-assisted puncture systems may encounter errors in lung biopsies where patient respiratory movement remains a significant interference factor [5]. Optical navigation robot-assisted puncture systems using optical markers have been proposed to track respiratory-related motion. However, their clinical applicability in human CT-guided lung biopsy remains limited and has not yet been fully validated [6]. Researchers have used head-mounted 3D-AR for CT-guided liver interventions on models, but they failed to simulate the patient’s breathing process [7]. To minimize the effects of respiratory motion, complex respiratory control strategies have also been reported in image-guided interventions. For example, Schullian et al. used general anesthesia with temporary disconnection of the endotracheal tube during CT-guided stereotactic liver biopsy to reduce respiratory motion and improve targeting accuracy. Although effective in that setting, such an approach requires general anesthesia and additional procedural resources, which may limit its routine clinical applicability, particularly for CT-guided percutaneous lung biopsy [8].

In clinical practice, some patients find it difficult to accurately understand and perform breath-holding maneuvers due to language barriers, hearing impairments, or a lack of prior experience. Furthermore, as most patients undergo the biopsy while awake, preoperative anxiety is common [9], which lowers pain tolerance and triggers physiological reactions such as elevated blood pressure and increased heart rate, potentially hindering the smooth implementation of the procedure. The rationale for this study was to determine whether syringe needle-assisted localization could permit CT-guided lung biopsy during natural tidal breathing without compromising procedural feasibility or safety. We hypothesized that the calm-breathing technique would achieve a high puncture success rate without an evident increase in procedure-related complications compared with conventional breath-holding. Accordingly, we addressed the following research question: Can CT-guided lung biopsy under calm breathing with syringe needle-assisted localization provide feasible procedural and safety outcomes compared with conventional breath-holding?

## Materials and methods

### Study population

This prospective, single-center, parallel-group randomized controlled trial enrolled 200 eligible patients who underwent CT-guided percutaneous lung biopsy at Ganzhou Cancer Hospital between January 2023 and October 2025. Before the biopsy procedure, participants were randomly assigned in a 1:1 ratio to either the syringe needle-assisted calm breathing group or the conventional breath-holding group. Each group included 100 participants. (Supplementary Figure 1) The calm breathing group consisted of 64 males and 36 females aged 21–78 years, whereas breath-holding group included 65 males and 35 females aged 33–79 years. Baseline demographic parameters, including age and sex, did not differ significantly between the groups. (Supplementary Table 1) All target pulmonary lesions included in this study had a maximum diameter greater than 15 mm on preprocedural CT imaging.

### Instruments

#### Consumables imaging equipment: philips brilliance 6 Spiral CT

Scanning parameters: 120 kV, 150 mA s/slice, slice thickness 3 mm, slice interval 3 mm. Biopsy System: coaxial guide needle (17 G, 15 cm); disposable cylindrical cutting biopsy needle (18 G, 15 cm, selectable sampling length of 16 mm or 8 mm; TSK Laboratory, Japan).

Localization Device: a self-made simple metal grid, constructed by cutting interventional metal guide wires into strips and fixing them in parallel with medical adhesive tape.

### Inclusion and Exclusion Criteria

Inclusion Criteria: 1) all lesions were located within the lung; 2) The puncture path did not cross the lung fissures; 3) No contraindications for puncture were present.

Indications: Normal preoperative results for complete blood count, coagulation function, and electrocardiogram (ECG).

All target lesions included in this study were solid pulmonary lesions identified on preprocedural CT imaging.

Exclusion Criteria (Contraindications): 1) Abnormal coagulation function, bleeding tendency, or recent use of anticoagulant/anti-angiogenic drugs [10]; 2) Severe emphysema; 3) Uncontrolled myocardial infarction or severe arrhythmia; 4) Uncontrollable severe cough.

### Biopsy procedure

A standardized preprocedural workup was completed, consisting of vital sign evaluation, ECG, laboratory testing of blood and coagulation parameters, assessment of liver and kidney function, glucose testing, and infection screening. Enhanced chest CT scans were reviewed. Patients were informed of the procedure and potential complication risks, and informed consent was signed. Patients in the breath-holding group underwent preoperative training: holding breath after a small inhalation.

#### Preoperative Preparation

1. An appropriate needle insertion position (supine, prone, or lateral) was selected; a vacuum cushion was used for fixation if patient cooperation was poor.
2. For elderly patients or those in poor physical condition, an ECG monitor was connected to monitor heart rate, blood pressure, blood oxygen, and respiration in real-time.
3. Participants were randomly assigned in a 1:1 ratio to either the calm breathing group or the breath-holding group before the biopsy procedure. The allocation sequence was generated in advance using a computer-generated random number table. The random sequence was generated and maintained by an investigator who was not involved in participant recruitment or biopsy procedures. The sequence remained concealed until the participant had been enrolled and group assignment was required. After allocation, the assigned respiratory coordination method was communicated to the procedural radiologist. Because of the evident differences between the two procedural techniques, blinding of the operators and participants was not feasible. Procedures in both groups were performed by two radiologists, each with more than 10 years of experience, according to the same standardized protocol.

#### Needle insertion operation

##### Experimental group

1. Attach a simple metal localization grid to the skin over the planned puncture area. Perform a local CT scan to identify the entry point, puncture path, and insertion angle using the laser alignment line and the corresponding grid marker. Measure the chest wall thickness to determine the appropriate depth of local anesthesia.
2. Disinfect and drape the puncture site. Infiltrate 2 % lidocaine layer by layer until adequate local anesthesia is achieved.
3. The patient maintained natural tidal breathing throughout the procedure. After local anesthesia, insert a 5-mL syringe needle (22 G, 32 mm) into the chest wall along the planned puncture path. Keep the needle tip outside the pleura to serve as a marker.
4. Repeat the CT scan using a limited scan range of 12–18 slices centered on the planned puncture level. Observe the vertical movement of the lesion during calm breathing. Reassess the final entry point and insertion angle according to the position of the syringe needle relative to the lesion.
5. Remove the syringe needle after confirming the puncture plan. Advance the coaxial needle step by step along the planned path. For lesions located in the lower lung field, when fine needle adjustment was necessary, the operator timed the adjustment to a naturally occurring end-expiratory phase observed during ongoing tidal breathing because lesion positioning was relatively stable at that moment. Patients were not instructed to pause breathing, prolong expiration, or alter their respiratory depth or rhythm. Thus, no voluntary breath-holding or patient-performed respiratory control maneuver was used. When the syringe needle marker and the target lesion showed an apparent positional mismatch on repeated CT images, the operator modified the planned entry point or puncture angle according to the observed relationship between the marker and the lesion.
6. Based on the lesion size and its distance from surrounding vessels, the heart, the trachea, and the pleura, the appropriate sampling length was determined. The biopsy needle was activated to the corresponding setting. The stylet of the guide needle was removed, and the biopsy needle was inserted for sampling. The tissue obtained was visually assessed. For each subsequent cut, the direction of the cutting groove and the angle of the puncture needle were slightly altered, obtaining 1–3 samples. After each sampling, the needle stylet was immediately reinserted to prevent bleeding from the needle sheath and to avoid air entering the lung or pleural cavity through the sheath.
7. After the procedure, the guide needle was removed, the puncture site was compressed, disinfected, and covered with sterile gauze.
8. A whole-chest CT scan was performed to check for complications (Supplementary Figure 2).
9. Throughout the calm-breathing procedure, patients maintained spontaneous tidal breathing and were not instructed to control their respiratory depth or rhythm or to perform any voluntary breath-hold. No dedicated respiratory-gating or respiratory-cycle monitoring device was used.

##### Control group

1. Breath-holding training: Patients were trained to hold their breath after a small inhalation; training was repeated until the performance was satisfactory.
2. CT localization: The method was identical to that used in the experimental group.
3. Disinfection, draping, and anesthesia: The procedures were the same as those used in the calm breathing group.
4. Puncture procedure: Instruct patients to hold their breath after a slight inhalation. Without syringe needle-assisted localization, advance the coaxial needle step by step according to the initial CT plan. Ask the patient to repeat the same breath-hold during each CT scan and needle adjustment until the needle reaches the target.
5. Sampling: Use the same sampling method as in the calm breathing group.
6. Post-procedure care: Remove the guide needle. Compress and disinfect the puncture site, then cover it with sterile gauze.
7. Postoperative imaging: Perform a full-chest CT scan to check for procedure-related complications, using the same protocol as in the calm breathing group.
8. During both CT image acquisition and needle advancement, instruct patients to hold their breath after a slight inhalation. Emphasize a consistent breath-hold throughout the procedure to reduce lesion movement.

For lesions in the lower lung field, both groups used a puncture path through a thinner part of the chest wall, although this resulted in a longer path through the lung (Supplementary Figure 3).

##### Postoperative management

Patients remained in bed for 4–6 h after the procedure. Those with pneumothorax were placed with the puncture side down. ECG monitoring continued for 24 h, and patients were followed for 1–5 days. If pneumothorax exceeded 30 % lung compression or massive hemorrhage occurred, treatment was based on the patient’s clinical condition. Options included closed chest drainage or consultation with the interventional radiology or thoracic surgery team.

### Data collection

The primary endpoint of this study was the puncture success rate, which was defined as successful acquisition of diagnostic tissue from the target lesion. Secondary endpoints included procedure-related complications (including pneumothorax, pulmonary hemorrhage, hemothorax, chest wall hematoma, and air embolism) and the number of needle adjustments.

Needle adjustments were recorded as the number of corrections made after the initial planned insertion. A limited CT scan (12–18 slices) followed each adjustment to confirm the needle position.

Puncture success rate: Determined by combining visual inspection of the specimen and pathological results. Unsuccessful cases included those where no tissue was obtained or where pathological results showed only normal alveolar tissue.

Complications: The incidence and severity of pneumothorax (defined as positive if lung collapse was ≥5 %), pulmonary hemorrhage, hemothorax, chest wall hematoma, and air embolism were recorded.

Calculation of pneumothorax: Pneumothorax was considered present when the lung compression ratio was ≥5 %. Among patients with pneumothorax, severity was classified as mild (≥5 % to <30 % lung compression), moderate (≥30 % to ≤50 %), or severe (>50 %). For measurement of the lung compression ratio, the total area of the ipsilateral thoracic cavity (A) was traced along the inner margin of the chest wall on the selected CT image, and the area occupied by pneumothorax (B) was traced on the same image. For relatively large pneumothoraces, measurements were performed at the level of the right or left pulmonary artery trunk, as appropriate. For localized pneumothoraces, measurements were performed on the central slice showing the air collection. The lung compression ratio was calculated using the following formula [11]:

Lung compression ratio=(B/A) × 100 %

Lesion location: The proportion of lesions located in the upper, middle, and lower lung fields was statistically recorded.

### Sample-size considerations

No formal *a priori* sample-size or power calculation was performed before participant enrollment. A total of 200 participants were enrolled during the study period, with 100 participants allocated to each group. Consequently, analyses of rare complications and other secondary outcomes were considered exploratory.

### Statistical analysis

All analyses were performed according to the originally assigned groups. Statistical analysis was performed using SPSS (version 22.0). Categorical variables are presented as frequencies and percentages. Between-group comparisons were performed using the chi-square test when its assumptions were satisfied; Fisher’s exact test was used for selected outcomes with extremely sparse event counts, including hemothorax, chest wall hematoma, and air embolism. Continuous variables are presented as mean±standard deviation (SD). Normality was assessed using the Shapiro–Wilk test. The number of needle adjustments was compared between the two groups using an independent-samples t-test. Although the distributions deviated from normality, the t-test was retained as the primary mean-based analysis because the two groups had equal and relatively large sample sizes and similar variances. A Mann–Whitney U test was additionally performed as a sensitivity analysis to evaluate the robustness of the result. The puncture success rate was considered the primary endpoint, whereas complication rates and the number of needle adjustments were considered secondary outcomes. For the number of needle adjustments, the between-group mean difference was calculated as the calm breathing group minus the breath-holding group. No adjustment for multiple comparisons was performed because the secondary analyses were exploratory. Effect estimates with 95 % confidence intervals were therefore considered together with p values.

### Ethics approval

The study was carried out in compliance with the ethical principles established by the institutional review board and in accordance with the Declaration of Helsinki (1964) and subsequent revisions.This study was approved by the Ethics Committee of Ganzhou Cancer Hospital (Approval No. (2022) KELUN Review (83)). Informed consent was obtained from all individuals included in this study, or their legal guardians or wards. This trial was not prospectively registered in a public clinical trial registry.

## Results

### Success rates

The results showed that tissue specimens were successfully obtained in 98 cases in the calm breathing group, with a success rate of 98 %. In the breath-holding group, 96 cases were successful, resulting in a success rate of 96 %. The success rates of the two groups were compared using the chi-square test, and no statistically significant difference was observed. (χ^2^=0.172, p=0.678). No statistically significant between-group difference in puncture success rate was detected (Table 1). The risk difference in puncture success rate between groups was 2.0 % (95 % CI, −2.7–6.7 %).

**Table 1:** Comparison of success rates.

Groups	n	Success, %	Failure, %	χ^2^	p-Value
Calm breathing group	100	98 (98)	2 (2)	0.172	0.678
Breath-holding group	100	96 (96)	4 (4)		

### Pneumothorax incidence

A total of 34 patients developed pneumothorax after the biopsy procedure. Pneumothorax occurred in 18 patients (18 %) in the calm breathing group and 16 patients (16 %) in the breath-holding group. No statistically significant between-group difference was detected in the overall incidence of pneumothorax (χ^2^=0.142, p=0.707). The risk difference, calculated as the calm breathing group minus the breath-holding group, was 2.0 % (95 % CI, −8.4–12.4 %) (Table 2).

**Table 2:** Comparison of pneumothorax incidence.

Outcome	Calm breathing group (n=100)	Breath-holding group (n=100)	Statistical value	p-Value
Any pneumothorax	18 (18)	16 (16)	χ^2^=0.142	0.707
No pneumothorax	82 (82)	84 (84)		
Mild pneumothorax	17 (17)	15 (15)		
Moderate pneumothorax	1 (1)	1 (1)		
Severe pneumothorax	0 (0)	0 (0)		

Data are presented as n (%). Pneumothorax was defined as a lung compression ratio of ≥5 %. Pneumothorax severity was classified as mild (≥5 to <30 %), moderate (≥30 to ≤50 %), or severe (>50 %). The Pearson chi-square test was used to compare the overall incidence of pneumothorax between the two groups. Among patients with pneumothorax, the distribution of mild versus moderate pneumothorax did not differ between the groups according to Fisher’s exact test (p=1.000). No severe pneumothorax occurred in either group.

Among the 18 patients with pneumothorax in the calm breathing group, 17 had mild pneumothorax (≥5 % to <30 % lung compression), and one had moderate pneumothorax (≥30 % to ≤50 %). Among the 16 patients with pneumothorax in the breath-holding group, 15 had mild pneumothorax, and one had moderate pneumothorax. No severe pneumothorax (>50 % lung compression) occurred in either group. The distribution of mild vs. moderate pneumothorax did not differ between the groups according to Fisher’s exact test (p=1.000).

The two patients with moderate pneumothorax, one in each group, remained clinically stable. Follow-up chest radiography or CT showed no progression, and neither patient reported clinically significant discomfort. Therefore, needle aspiration and closed chest drainage were not required. Overall, most pneumothoraces were mild, and no statistically significant difference in pneumothorax incidence was detected between the two respiratory coordination methods.

### Pulmonary hemorrhage incidence

Pulmonary hemorrhage was defined as intraparenchymal bleeding, excluding minor band-like oozing along the needle track. In this study, pulmonary hemorrhage occurred in 3 cases (3 %) in the calm breathing group and 5 cases (5 %) in the breath-holding group, with no statistically significant difference between the two groups (χ^2^=0.130,p=0.718). Only one case involved extensive alveolar hemorrhage, while the others were patchy alveolar hemorrhages; no cases of massive hemoptysis occurred (Figure 1). No patients experienced dyspnea, worsening hemorrhage, or required interventional hemostasis. This indicates that under the conditions of this study, pulmonary hemorrhage was a mild and controllable event. No statistically significant between-group difference in pulmonary hemorrhage incidence was detected (Table 3).

**Figure 1:**
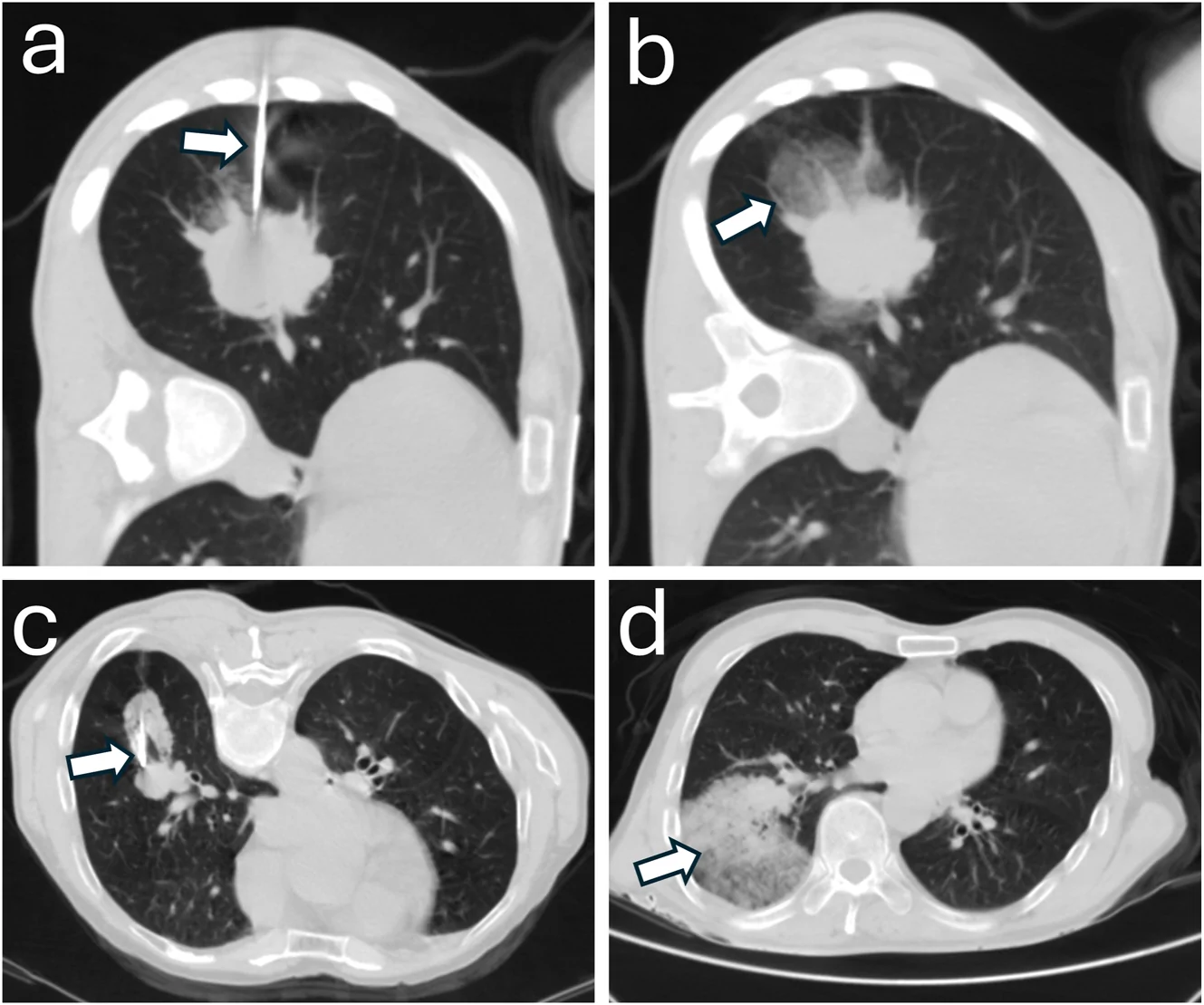
Representative CT images demonstrating pulmonary hemorrhage following CT-guided percutaneous lung biopsy. (a, b) Images from Patient 1, who underwent biopsy of a right lower lobe lesion in the left lateral decubitus position. (a) CT image obtained immediately after biopsy. (b) Patchy alveolar hemorrhage surrounding the needle tract. (c, d) Images from Patient 2, who underwent biopsy of a right lower lobe lesion in the prone position. (c) CT image obtained immediately after biopsy. (d) Extensive alveolar hemorrhage along the needle tract. The patient was subsequently repositioned to the supine position after withdrawal of the biopsy needle. Open arrows indicate the areas of pulmonary hemorrhage.

**Table 3:** Comparison of pulmonary hemorrhage incidence.

Groups	n	Positive, %	Negative, %	χ^2^	p-Value
Calm breathing group	100	3 (3)	97 (97)	0.130	0.718
Breath-holding group	100	5 (5)	95 (95)		

### Hemothorax incidence

Hemothorax occurred in two patients in the calm breathing group and three patients in the breath-holding group, for a total of 5 cases. A total of five patients in both the calm breathing and breath-holding groups were found to have a small amount of intrapleural blood, manifesting as arc-shaped high-density shadows in the pleural cavity. All cases were trace or minor, and follow-up CT scans within 30 min showed no increase in the volume of hemorrhage. Fisher’s exact test showed no statistically significant between-group difference (p=1.000). The source of bleeding was primarily attributed to needle track injury of the lung tissue or soft tissue hemorrhage of the chest wall. The hemothorax did not progress, and no patients reported significant symptoms such as chest pain or tightness, suggesting these were mild, self-absorbing hemorrhages. No statistically significant between-group difference in hemothorax incidence was detected (Table 4).

**Table 4:** Comparison of hemothorax incidence.

Groups	n	Positive, %	Negative, %	p-Value
Calm breathing group	100	2 (2)	98 (98)	1.000^a^
Breath-holding group	100	3 (3)	97 (97)	

^a^Fisher’s exact test.

### Chest wall hematoma incidence

Chest wall hematoma results from hemorrhage of small vessels in the chest wall due to puncture injury. In this study, only one case of chest wall hematoma occurred in the control group. The hematoma was small (approximately 13 × 22 × 15 mm). It remained stable after 10 min of manual compression at the puncture site, and no further treatment was required. Chest wall hematoma was uncommon in both groups, indicating that chest wall hematoma was rare, and no statistically significant between-group difference was detected (Table 5).

**Table 5:** Comparison of chest wall hematoma incidence.

Groups	n	Positive, %	Negative, %	p-Value
Calm breathing group	100	0 (0)	100 (100)	1.000^a^
Breath-holding group	100	1 (1)	99 (99)	

^a^Fisher’s exact test.

### Incidence of air embolism

In the calm breathing group, one patient developed a small punctate air embolism in the left ventricular apex after the procedure. The patient remained in the supine position and was closely monitored. No symptoms developed during the following days, and no serious events, such as myocardial infarction or cerebral infarction, occurred (Table 6).

**Table 6:** Comparison of air embolism incidence.

Groups	n	Positive, %	Negative, %	p-Value
Calm breathing group	100	1 (1)	99 (99)	1.000^a^
Breath-holding group	100	0 (0)	100 (100)	

^a^Fisher’s exact test.

All cases of pneumothorax were treated with supplemental oxygen, ECG monitoring, and clinical observation. None required needle aspiration or chest tube drainage. Pulmonary hemorrhage occurred in three patients in the calm breathing group and five patients in the breath-holding group. Only one patient in the calm breathing group developed extensive alveolar hemorrhage and received intravenous hemocoagulase. The remaining patients recovered without additional treatment.

Hemothorax was minimal or small in all five cases. Repeat CT within 30 min showed no increase in the amount of pleural blood. All patients received observation and ECG monitoring only. No patient required hemostatic drugs, transcatheter embolization, or chest tube drainage. One patient developed a small chest wall hematoma. The hematoma remained stable after 10 min of manual compression, and no further treatment was needed. The patient with systemic air embolism recovered after oxygen therapy, ECG monitoring, and one day of bed rest in the supine position. Follow-up CT showed complete resolution of the intravascular air. Hyperbaric oxygen therapy was not required, and no neurological or cardiovascular sequelae were observed.

### Needle adjustments

The number of needle adjustments was recorded after the initial needle insertion. The mean number of adjustments was 1.82±1.59 in the calm breathing group and 2.25±1.73 in the breath-holding group. The mean difference, calculated as the calm breathing group minus the breath-holding group, was −0.43 adjustments (95 % CI, −0.89 to 0.03; t=−1.83, p=0.068), indicating no statistically significant difference in the primary mean-based analysis (Table 7). Because the distributions were non-normal, a Mann–Whitney U test was additionally conducted as a sensitivity analysis and yielded U=4,212 and p=0.047. Thus, the calm breathing group showed a tendency toward fewer needle adjustments, but the statistical interpretation was sensitive to the analytical method used.

**Table 7:** Comparison of needle adjustments between the two groups.

Variable	Calm breathing group (n=100)	Breath-holding group (n=100)	Mean difference (95 % CI)	t	p-Value
Needle adjustments	1.82 ± 1.59	2.25 ± 1.73	−0.43 (−0.89 to 0.03)	−1.83	0.068

Data are presented as mean ± standard deviation. The mean difference was calculated as the Calm Breathing Group minus the breath-holding group. The independent-samples *t*-test was used as the primary analysis to compare the mean number of needle adjustments between the groups. Because the data were not normally distributed, a Mann–Whitney U test was additionally performed as a sensitivity analysis, yielding U=4,212 and p=0.047. The sensitivity result should be interpreted cautiously because the number of needle adjustments was a secondary outcome and no adjustment for multiple comparisons was performed.

### Proportional distribution across lung fields

The lower lung field exhibits the greatest magnitude of respiratory motion, which could theoretically increase the difficulty and complication risk of CT-guided biopsy during calm breathing. In this study, the proportion of cases involving the lower lung field was comparable between the two groups. Lung fields were demarcated by horizontal lines at the lower margins of the second and fourth anterior ribs. Similar distributions of lesions in the upper, middle, and lower lung fields were noted in both groups, without significant between-group differences (Table 8). Stratified analyses of puncture success and individual complications according to lung field were not performed because the event counts within each subgroup were insufficient for reliable estimation.

**Table 8:** Comparison of lesion distribution in upper, middle, and lower lung fields.

Groups	n	Upper field, %	Middle field, %	Lower field, %
Calm breathing group	100	22 (22)	38 (38)	40 (40)
Breath-holding group	100	25 (25)	40 (40)	35 (35)
χ^2^		0.250	0.084	0.533
p-Value		0.617	0.772	0.465

Data are presented as n (%). Each χ^2^ statistic and p value represents a separate Pearson chi-square comparison of the proportion of lesions located in the specified lung field between the two groups. All expected cell counts were <5.

The risk differences between the calm breathing group and the breath-holding group were 2.0 % (95 % CI, −8.4–12.4 %) for pneumothorax, −2.0 % (95 % CI, −7.4–3.4 %) for pulmonary hemorrhage, −1.0 % (95 % CI, −5.3–3.3 %) for hemothorax, −1.0 % (95 % CI, −3.0–1.0 %) for chest wall hematoma, and 1.0 % (95 % CI, −1.0–3.0 %) for air embolism.

## Discussion

This study addressed whether CT-guided percutaneous lung biopsy under calm breathing with syringe needle-assisted localization could provide feasible procedural and safety outcomes compared with conventional breath-holding. High puncture success rates were observed in both groups, and no statistically significant between-group differences were detected in puncture success or procedure-related complication rates, supporting the feasibility of the calm-breathing technique in solid pulmonary lesions larger than 15 mm. However, because this study was not designed as an equivalence or non-inferiority trial, the absence of statistically significant differences should not be interpreted as proof of equivalence.

According to the Analysis Report on the Epidemiological Characteristics of Malignant Tumors in China (2022) released by the National Cancer Center of China, lung cancer ranks first among malignant tumors in China in terms of both incidence and mortality [12]. Pathological examination remains the gold standard for distinguishing benign from malignant pulmonary lesions. CT-guided percutaneous lung biopsy provides an effective way to obtain tissue samples and has high diagnostic accuracy. It is now widely used as a minimally invasive procedure and serves as an important complement to bronchoscopic biopsy for diagnosing pulmonary lesions. A recent study of 442 cases reported that the diagnostic accuracy, sensitivity, and specificity of CT-guided percutaneous lung biopsy were 97.6 %, 97.3%, and 100 %, respectively [13]. Even for ground-glass opacity (GGO) lesions ≤ 20 mm, the diagnostic accuracy reaches 90.4 % [14].

Despite continuous refinements in diagnostic and puncture techniques, successful completion of the procedure and tissue acquisition remain challenging for some patients due to individual physiological differences and varying levels of cooperation. *Montaudon* et al. reported a higher false-negative rate for small lesions with a diameter ≤10 mm, confirming that lesion size is a significant factor influencing false-negative results [15]. A South Korean multicenter study also identified a lesion size ≤ 1 cm as an independent risk factor for diagnostic failure [16]. Because all included lesions were solid and larger than 15 mm, the findings should not be extrapolated directly to subsolid nodules, ground-glass opacities, or very small pulmonary lesions.

Respiratory motion involves both inspiration and expiration. During inspiration, the thoracic cavity expands in the anteroposterior, transverse, and vertical directions. During expiration, the chest wall contracts and the lungs recoil, causing the lung lesion to move. In routine CT-guided percutaneous lung biopsy, patients are usually asked to hold their breath after a slight inhalation. Before the procedure, radiologists explain the importance of breath holding and provide brief training [17]. The goal is to keep lesion localization and needle insertion in the same respiratory phase, such as end-inspiration or end-expiration.

Although this approach can reduce lesion movement, many patients cannot maintain the same breath-holding depth throughout the procedure because of pain, anxiety, or poor cooperation. Some patients also have difficulty following instructions because of hearing impairment or language barriers. In conventional CT-guided biopsy, the needle is advanced between CT scans rather than under real-time imaging. If the breathing pattern changes, the lesion may move away from the planned needle path. When this happens, the needle may enter normal lung tissue instead of the target lesion, requiring withdrawal and repeated adjustment or reinsertion [18]. Therefore, breath-holding techniques alone cannot completely overcome poor respiratory cooperation, which remains an important cause of puncture difficulty.

*Dasom Kim* et al. retrospectively analyzed differences in percutaneous lung biopsy outcomes among patients with varying respiratory amplitudes under calm breathing, showing that cases with larger respiratory amplitudes faced increased puncture difficulty [19]. A recent study suggested that using opioid analgesics can weaken the central respiratory drive, leading to smoother breathing and reduced pneumothorax complications [20]. While a prone position can moderately reduce lung mobility, not all patients are suitable for this position.

Pneumothorax and pulmonary hemorrhage are common complications [21]. The incidence of pneumothorax in CT-guided non-coaxial cutting biopsy is reported at 27 % [22]. Recent large-scale data indicates that the pneumothorax rate for CT-guided percutaneous coaxial lung biopsy is 27.3 %, with small target volume, emphysema, and deep-seated lesions being significant risk factors [23]. The risk is particularly elevated in patients with comorbid COPD [24]. Some research suggests that forced expiration followed by breath-holding before needle withdrawal can nearly halve the incidence of pneumothorax [25]. Other complications include hemoptysis, intercostal artery injury, internal mammary artery injury, and air embolism; the latter is the most severe, potentially leading to cerebral or myocardial infarction [26]. Another study found that 22.5 % (326/1,452) of patients developed pneumothorax, with massive pneumothorax often associated with punctures traversing the lung fissures [27].

Respiratory motion may have a greater impact on the biopsy of small pulmonary lesions because of their limited target size. Previous studies have reported lower diagnostic accuracy for pulmonary nodules ≤10 mm compared with larger lesions [17]. However, the present study was not designed to specifically evaluate small pulmonary nodules. To reduce respiratory influence, *Enrique Carrero* et al. described two cases using ultrasound-guided phrenic nerve blocks to paralyze the ipsilateral diaphragm during CT-guided fine-needle aspiration; however, this carries risks of Horner’s syndrome and inadvertent anesthesia of the adjacent brachial plexus [28].

Several studies have attempted to reduce the effect of respiratory motion during CT-guided lung biopsy. *Ha* et al. has proposed a dual-needle approach to address respiratory-related motion. In this technique, one needle is maintained as a positional reference or fixation point, while a second needle is used to complete the biopsy. A previous study reported a 100 % success rate among 17 patients; however, the small sample size limits the strength of evidence, and further studies with larger cohorts are required to confirm its clinical benefit [18]. *Ji* et al. [29] reported a planar puncture template for small pulmonary nodules. In their study, 34 of 56 patients required two fixation needles and 13 required one fixation needle to reduce respiratory motion.

Respiratory-related lesion movement has also been measured with four-dimensional CT (4D-CT). Although these studies focused on radiotherapy planning rather than lung biopsy, they showed that pulmonary lesions can move considerably during respiration. This suggests that lesion excursion during calm breathing is usually limited to approximately 1.0 cm, apart from small lower-lung lesions subject to diaphragmatic influence [30]. These findings provide useful background for understanding the effect of respiratory motion during CT-guided biopsy. Conversely, a study on preoperative coil localization for lung nodules showed that when the needle is inserted during calm breathing, the success rate of CT-guided puncture is independent of respiratory amplitude [31].

Several techniques have been developed to improve the accuracy of CT-guided lung biopsy, including dual-needle localization, laser guidance, and robotic-assisted navigation. These techniques may improve targeting accuracy in selected patients but usually require additional equipment, specialized training, or higher procedural costs. Syringe needle-assisted localization uses materials already available in routine clinical practice and does not require additional hardware. The technique is simple to perform, inexpensive, and may be particularly useful in resource-limited centers. In this study, syringe needle-assisted localization involved inserting a syringe needle into the chest wall after local anesthesia, following the planned direction and angle, without reaching the pleura. The syringe needle served as a visual reference to evaluate the relative positional relationship between the planned entry point and the lesion during calm breathing. The operator assessed whether the projected puncture trajectory remained aligned with the target lesion during respiratory motion and adjusted the puncture angle when necessary. Syringe needles are lightweight and easily fixed even in thin chest walls, allowing clinicians to adjust the direction as needed. Using a 17G guide needle for localization increases the risk of injuring chest wall vessels or requires deeper insertion for stabilization due to its weight, which risks damaging deep tissues or the pleura. One report mentioned that for patients with thin chest walls, assistant manual fixation (requiring protective gear) was necessary when using non-coaxial needles for localization [32]. Furthermore, chest wall syringe needle-assisted localization prevents severe errors, such as forgetting to move the CT table to the planned puncture level or the patient moving before the entry point is marked. This method entirely avoids such incidents, reducing unnecessary injury. We also used a self-made localization grid during procedural planning. The grid provided a simple and low-cost way to mark the skin surface and plan the puncture path. Together with syringe needle-assisted localization, it helped identify the entry point more accurately and made the procedure easier to perform. However, this grid has not been compared with commercially available localization systems. Its reproducibility in different centers also remains unclear. Further multicenter studies are needed to evaluate its performance and develop standardized protocols for wider clinical use.

Lesions in the lower lung field are heavily influenced by respiration, even in the breath-holding control group. To facilitate needle adjustment, we preferred a thinner chest wall approach and a path traversing more normal lung tissue for these lesions, as a longer path through lung tissue provides better “anchoring” and space for correction [33]. For superficial lesions, a tangential approach was used to stabilize the guide needle and reduce the risk of pleural seeding [34]. For small lesions at the lung base where respiratory amplitude was large, the experimental group performed needle adjustments at the end of expiration. All lesions included in this study were solid pulmonary lesions. Therefore, the findings may not be directly applicable to subsolid nodules or ground-glass opacities, which may present different technical challenges during CT-guided biopsy. In addition, lesion size was not systematically analyzed and may represent a potential confounding factor affecting procedural outcomes and complication rates. Therefore, the present findings may not be directly applicable to very small pulmonary lesions, in which respiratory motion may have a greater influence on targeting accuracy and procedural complexity. Future studies should evaluate the performance of this technique in smaller lesions and perform stratified analyses according to lesion size.

The calm breathing group showed a numerically lower mean number of needle adjustments than the breath-holding group. However, the primary independent-samples t-test did not reach statistical significance, whereas the Mann–Whitney U sensitivity analysis yielded a marginally significant result. This secondary finding was therefore sensitive to the analytical method and should be interpreted cautiously. The syringe needle may provide a visual reference for adjusting the puncture trajectory before pleural entry, but larger studies are required to determine whether the technique consistently reduces the number of needle adjustments.

Several limitations should be acknowledged. First, this was a single-center trial, and operator blinding was not feasible because the two procedures were visibly different. Nevertheless, both radiologists followed the same standardized protocol, and the study outcomes were defined in advance. Second, no formal *a priori* sample-size calculation was performed, the trial was not prospectively registered, and no multiplicity adjustment was applied to the secondary outcomes. Therefore, the possibility of type II error for small or rare-event differences and type I error across multiple secondary comparisons cannot be excluded; the secondary findings should be interpreted as exploratory rather than as evidence of superiority or equivalence. Third, respiratory motion and marker–lesion displacement were assessed qualitatively rather than quantitatively, and patient-reported outcomes such as anxiety, pain, procedural comfort, and satisfaction were not systematically collected. Finally, all included lesions were solid and larger than 15 mm, subgroup event counts were insufficient for reliable stratified outcome analyses, and long-term follow-up data were unavailable. Larger, prospectively registered multicenter trials with prespecified sample-size calculations and quantitative respiratory-motion assessment are warranted.

## Conclusions

CT-guided percutaneous lung biopsy under calm breathing using syringe needle-assisted localization was feasible in this study of solid pulmonary lesions larger than 15 mm. No statistically significant between-group differences were detected in puncture success or procedure-related complication rates. The technique may provide an alternative when adequate breath-holding cooperation is difficult; however, its applicability to very small lesions, basal lesions, and lesions with marked respiratory excursion remains uncertain. The possible reduction in needle adjustments was exploratory and requires confirmation in larger, prospectively registered randomized trials.

## Supplementary Material

Supplementary Material
