# Supplementary material for: CT-guided percutaneous lung biopsy under calm breathing
Source: Open Med (Wars). 2026 Nov 2;21(1):20261533. doi: 10.1515/med-2026-1533 (PMC13725329; doi:10.1515/med-2026-1533)
Supplement: Supplementary file 1 [file j_med-2026-1533_suppl_001.docx]

**Supplementary Table 1**: Baseline Patient Characteristics.

|  | Experimental Group  (n=100) | Control Group (n=100) | t/χ² value | *P* value |
| --- | --- | --- | --- | --- |
| Age | 60.16±11.09 | 60.2±10.86 | t = -0.026 | 0.979 |
| Male  Female | 64  36 | 65  35 | χ²＝0.022 | 0.883 |

Supplementary Figure 1. CONSORT participant flow diagram

Supplementary Figure 2. (a, b) Display of the body surface localization grid. (c) Localization using a syringe needle positioned within the chest wall. (d) Initial insertion of the coaxial guide needle, having penetrated the pleura. (e) Adjustment of the needle to the vicinity of the lesion. (f) The guide needle has successfully reached the target.

White arrows indicate the syringe needle used as the localization marker.

Supplementary Figure 3. (a, b, c, d) Sequential needle insertion for lesions in the lower lung field. The needle was inserted through a thinner section of the lateral chest wall to facilitate the adjustment of the insertion angle. The increased distance between the entry point and the lesion allows for multiple needle adjustments and better stabilization of the lung tissue.
